# Inducing Deep Sweeps and Vortex Ejections on Patterned Membrane Surfaces to Mitigate Surface Fouling

**DOI:** 10.3390/membranes14010021

**Published:** 2024-01-13

**Authors:** August H. Young, Nico Hotz, Brian T. Hawkins, Zbigniew J. Kabala

**Affiliations:** 1 Duke Center for WaSH-AID, Durham, NC 27701, USA; bthawkin@ncsu.edu; 2 Mechanical Engineering and Materials Science, Duke University, Durham, NC 27710, USA; nico.hotz@duke.edu; 3 Electrical and Computer Engineering, Duke University, Durham, NC 27710, USA; 4 Civil and Environmental Engineering, Duke University, Durham, NC 27710, USA; zbigniew.kabala@duke.edu

**Keywords:** filtration, concentration polarization, fouling mitigation, surface pattern, pulsed flow, deep sweep, vortex ejection

## Abstract

Patterned membrane surfaces offer a hydrodynamic approach to mitigating concentration polarization and subsequent surface fouling. However, when subjected to steady crossflow conditions, surface patterns promote particle accumulation in the recirculation zones of cavity-like spaces. In order to resolve this issue, we numerically subject a two-dimensional, patterned membrane surface to a rapidly pulsed crossflow. When combined with cavity-like spaces, such as the valleys of membrane surface patterns, a rapidly pulsed flow generates mixing mechanisms (i.e., the deep sweep and the vortex ejection) and disrupts recirculation zones. In only four pulses, we demonstrate the ability of these mechanisms to remove over half of the particles trapped in recirculation zones via massless particle tracking studies (i.e., numerical integration of the simulated velocity field). The results of this work suggest that when combined with a rapidly pulsed inlet flow, patterned membrane surfaces can not only alleviate concentration polarization and the surface fouling that follows but also reduce the need for traditional cleaning methods that require operational downtime and often involve the use of abrasive chemical agents.

## 1. Introduction

### 1.1. Challenges to Membrane Operation

Membrane filtration is a popular tool in treating wastewater for reuse [1,2,3]. Compared to other treatment technologies, membranes require less energy than thermal treatment processes and less time than traditional filtration methods (e.g., sand filtration). Further benefits of membrane technologies are modest footprints, relatively low capital costs, and a demonstrated efficacy in producing pathogen-free outputs. However, in application to complex waste streams with high fouling potential, membranes face significant operational challenges, namely concentration polarization and surface fouling.

Although concentration polarization is a reversible phenomenon (often mitigated via disturbance of the solute layer at the membrane surface [4]), it leads to fouling of the membrane surface when left uncurbed [5,6]. This type of fouling significantly thwarts the wide-scale adoption of nanofiltration [7] and poses similar operational challenges to the implementation of microporous membranes (i.e., microfiltration and ultrafiltration modules) [4]. The overwhelming consensus is that these phenomena pose the most significant hurdles in the application of pressure-driven membrane processes [8,9,10,11,12].

Fouling at the membrane surface is typically diminished by chemical modification of the membrane surface or cleaning procedures. The former is undoubtedly beneficial to the targeting and rejection of certain contaminant species but is cautioned by authors who express concerns about stability, longevity, and effect on membrane performance [10,13,14]. Additionally, the latter is routinely used to restore membrane performance but is also associated with several significant drawbacks. Chemicals commonly used in cleaning procedures can deteriorate the physical properties of the membrane surface, reducing selectivity to solutes in the feed stream [15], especially in the case of polymeric modules [16] and other thin film composites [17]. Membranes can also be damaged by incorrect chemical cleaning procedures [18], and one must consider the negative environmental impacts of chemical use [19]. At the very least, operational downtime should also be considered a drawback to any traditional cleaning method that requires taking the membrane offline [12]. Thus, it is advantageous to minimize the need for and frequency of chemical cleanings.

### 1.2. Hydrodynamic Solutions

In order to postpone the need for chemical cleaning agents, it is necessary to mitigate particle aggregation at the membrane surface—the precursor to membrane fouling. In the following sections, we review two commonplace methods by which researchers attempt to keep particulates in the bulk flow: hydrodynamic manipulation of the feed flow and patterned membrane surfaces.

#### 1.2.1. Manipulation of the Feed Flow

Reducing the severity of concentration polarization requires that the solute layer at the membrane surface be disturbed. Most commonly, this is achieved by operating the membrane in crossflow in the transitional/turbulent flow regime to increase wall shear and induce local turbulence; it is widely recognized that low feed Reynolds numbers result in increased concentration polarization [20,21]. This technique, however, can be cost-prohibitive due to the associated energy demand of feed pumps [10,21]. Other commonplace approaches in the crossflow operational mode include limiting the length of the membrane surface (to inhibit boundary layer growth) and utilizing pulsatile flows, flow reversals, and centrifugal instabilities [22]. Jaffrin [23] provides a thorough overview of these techniques and highlights their ability to reduce surface fouling and improve filtration performance. Recent examples of studies that highlight the benefit of pulsed flow over steady include Kürzl and Kulozik [24], Liu et al. [25], and Wang et al. [26]. In another related example, Echakouri et al. [27] use a periodic feed pressure technique to minimize surface fouling.

Although there are a limited number of investigations into the dynamics of pulsed flow in membrane channels with spacers (e.g., see [24,28,29,30]), there are none that combine the study of pulsed flow with patterned membrane surfaces. While both research endeavors involve the simulation of vortex dynamics, the former focuses on the efficacy of using vortex shedding to scour the length of an otherwise flat membrane surface.

#### 1.2.2. Manipulation of the Membrane Surface

Patterned membrane surfaces, which induce local turbulence and high surface shear, offer yet another hydrodynamic approach to mitigating concentration polarization and surface fouling. Multiple research groups have demonstrated the ability to create micro- and nano-structures on flat and tubular membrane surfaces. Among them, we highlight Won et al. [31,32,33], who demonstrated successful fabrication of membrane surfaces with pyramids and prism patterns; in our work, we replicate the two-dimensional surface pattern utilized by Won et al. [32]. Heinz et al. [34] provide a comprehensive list of thirty-five studies concerning the fabrication of patterned polymeric membrane surfaces used in separation applications. Barambu et al. [35] summarize the approaches used to produce these patterns and the subsequent effect on membrane performance. Chauhan et al. [36], Zare and Kargari [12], and Ibrahim and Hilal [37] provide more recent and nearly concurrent review articles.

Aside from the recent study of Ward et al. [38], there is ample numerical and experimental evidence that patterned membrane surfaces effectively mitigate concentration polarization. Çulfaz et al. appear to be among the first to study the effect of a controlled surface pattern on fouling and membrane performance, excluding previous work on corrugated membranes, as summarized by Ibrahim and Hilal [37]. There was, however, ample interest in the study of spacer patterns in cross-flow channels beforehand (e.g., see Ma and Song [39] for results and a brief summary of prior studies). In their study on the fouling behavior of micro-structured hollow fibers in the filtration of sodium alginate, Çulfaz et al. [11,40] found that, in comparison to smooth fibers, the structured fibers exhibited a higher degree of reversibility in surface fouling. The authors attribute this phenomenon to a looser packing of the deposited particles onto the structured membrane, which is ultimately more conducive to removal procedures. Similar results were obtained by Rickman et al. [41], who found that patterned membranes recover more of their initial flux than their nonpatterned counterparts. Further, Ward et al. [42] found that when exposed to feed streams containing E. coli cells, nanopatterned membranes recovered 18% more of their initial flux than non-patterned membranes. In this case, the improved cleanability of the patterned membrane is attributed to the fact that the E. coli cells cannot deposit into the valleys of the membrane, thus thwarting the rate of biofouling in these regions [42].

Following the work of Çulfaz et al. [11,40], Won et al. [33] utilized a new patterning process to treat wastewater in crossflow; they found that the deposition of microbial cells onto the patterned membrane surface was significantly reduced. The authors attribute the reduction to the apexes of the surface pattern, which they deem responsible for inducing local turbulence and high shear—a conclusion also supported by their later work on the biofouling of prism patterns [31]. Shortly after the work of Won et al. [33], Lee et al. [43] published similar findings, indicating the importance of flow characteristics and shear stress distribution for the frequency and severity of particle deposition in the valleys of the surface pattern. Shang et al. [7] later confirmed that the reduction in concentration and thickness of the concentration polarization layer is attributed to the increased shear generated by the surface pattern.

In another study on triangular-patterned surfaces, Choi et al. [44] highlighted a dependence on the size of the particles in the feed suspension and pore water flux in addition to the crossflow feed rate. The authors found that, for the microfiltration of mixed suspensions (i.e., those with a large distribution in particle size), the deposition of larger particles affected the flow streamlines and therefore the deposition of smaller particles. They also found that the bulk flow and vortex streamlines were “well-separated” from one another, making it difficult for small particles to traverse the separation and deposit onto the valleys of the membrane surface. Malakian and Husson [45] recently used this argument to explain the low levels of protein deposition they observed in the valleys of a herringbone surface pattern. Jung et al. [46] found that the particles they studied tended to deposit into the surface valleys and not at the peaks, indicating a conclusion later drawn by Jung and Ahn [47]: a patterned membrane surface can tremendously reduce surface fouling, given a judicious choice in Reynolds number and pattern depth relative to average particle size. Kim et al. [48] recently offered further confirmation of these dependencies via an alternative approach (i.e., a herringbone-patterned mixer to induce chaotic advection in a flat sheet membrane module).

Won et al. [32] provided a numerical investigation of patterned membrane surfaces, manipulating the parameters of the surface pattern to determine the effect on particle deposition. The authors showed that the tested patterns yielded a significant reduction in the mass attached to the membrane wall. Maruf et al. [10] and Jamshidi Gohari et al. [9] demonstrated a decrease in surface fouling accompanied by an increase in orientation angle between the surface pattern lines and feed flow direction. Malakian et al. [49] highlighted yet another geometric dependence on pattern width, noting the subsequent effect on vortex size and the ability of the vortex to shield the membrane surface from particle deposition (a phenomenon previously termed “vortex-induced shielding” by Choi et al. [50]). Wang et al. [51] provided a thorough summary of the work that has been undertaken, to date, on correlating pattern configuration with the rate of particle deposition and fouling, both numerically and experimentally. Again, we note that the summarized works focused only on a steady inlet flow condition.

### 1.3. Research Opportunity

Although there is extensive documentation that patterned membrane surfaces reduce the thickness and degree of concentration in the concentration polarization layer, surface patterns produce stationary vortices in valleys and cavity-like spaces capable of trapping buoyant particles. It is recognized that the presence of these stagnant zones promotes particle aggregation and induces surface fouling in the pattern valleys [8,43,46,51,52] and can thus result in an overall higher degree of fouling [12]; the same is true for studies concerning the fouling of membrane channels with spacers. Wang et al. [51] explain that while vortices can create flow separation in pattern valleys and therefore hinder particle deposition along the valley surface, they can also capture foulants. Once captured, foulants generally remain and aggregate in the recirculation zone [52], given the need to traverse sometimes significant flow separations to move back into the bulk flow [32]. For example, in their study of a rectangular surface pattern, Gençal et al. [8] found that an increase in surface pattern height relative to pattern width created larger dead zones in the pattern bottoms and exacerbated surface fouling. Given the tendency of trapped particles to aggregate and aggravate surface fouling in pattern valleys, there remains the need to flush these stagnant zones with a comparatively clean flow volume; thus, we look to the vortex ejection and deep sweep mechanisms. Because we can induce these mechanisms during processing, they are attractive alternatives to traditional cleaning methods (i.e., clean water flushes and chemical cleanings) that require operational downtime and often subject membrane surfaces to degradation.

**Figure 1:**
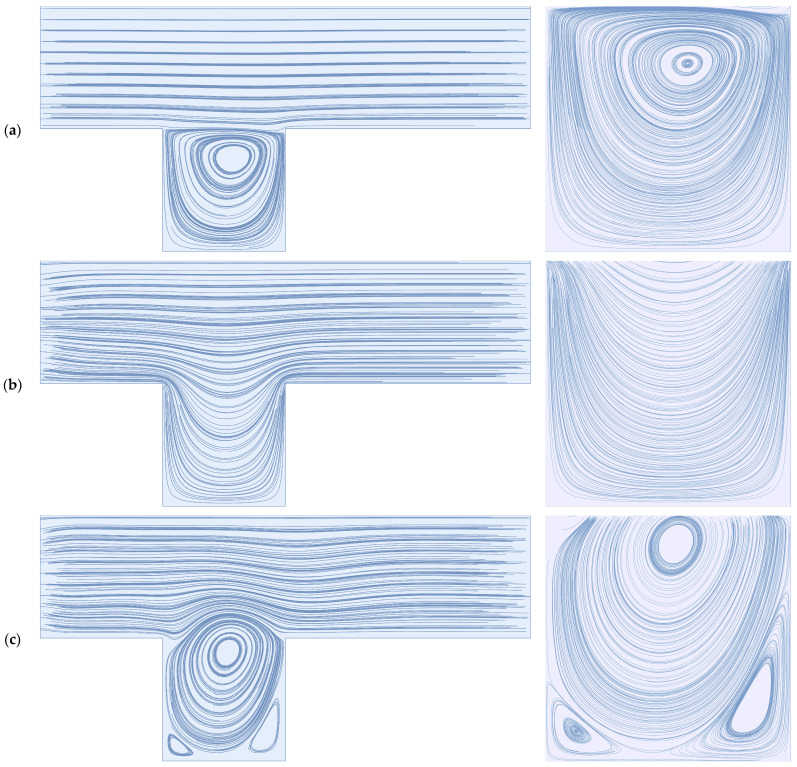
(**a**) A stationary cavity vortex driven by a steady feed flow with a Reynolds number of 100; (**b**) the deep sweep mechanism resulting from a sudden increase in flow volume; (**c**) the vortex ejection mechanism resulting from a sudden decrease in flow volume.

The deep sweep and vortex ejection mechanisms are generated by rapidly pulsed flow—a sudden decrease in feed flow volume results in an ejection of the cavity vortex, while a sudden increase in feed flow volume results in a deep sweep of the feed flow into the cavity space. We illustrate these mechanisms below for the dead-end pore model, which is discussed extensively by Young and Kabala [53] and is standard in the study of impermeable surfaces with patterns and groves, e.g., Shen and Floryan [54] and Fang et al. [55]. To generate these mechanisms, we utilize a sinusoidal waveform with an average value of 0.5, an amplitude of 0.5, and a frequency of 0.25. As we discuss in Section 2 (Materials and Methods), all simulations are dimensionless. The average flow Reynolds number for this simulation is 100. For comparison, we also illustrate a stationary cavity vortex driven by a steady feed flow. There is a no-slip and no-penetration condition applied to the upper and lower boundaries of the simulation domain pictured on the left-hand side of Figure 1; the flow direction is from left to right. Readers are referred to the Supplemental Material (DEP_CavityAnimation.mp4) to observe these mechanisms in sequence.

Previously, Kahler and Kabala [56,57,58] found that these mechanisms are responsible for enhanced transport between cavity spaces and the bulk flow; they build upon the work of Sobey [59], who determined that enhancement was a result of vortex emptying and filling in cavity-like spaces after Bellhouse et al. [60] designed a pulsatile flow system to create unsteady vortices and enhance gas transport in furrowed channels. In simulation, Kahler and Kabala [56] found that rapidly pulsed pumping in the dead-end pore space recovered 11% more contamit than steady flow. They also found that rapidly pulsed pumping recovered the same amount of contaminant as steady flow seven times faster. Later, Kahler and Kabala experimentally verified accelerated removal in column testing on granular media [58]. Therefore, it is plausible to suspect that rapidly pulsed pumping could disrupt concentration polarization at the membrane surface via the deep sweep and the vortex ejection mechanisms. Given the ability of these mechanisms to move otherwise trapped particles back into the bulk flow, a rapidly pulsed feed flow could significantly reduce the fouling of patterned membrane surfaces. Thus, we use this work as an opportunity to subject a patterned membrane surface to a rapidly pulsed inlet flow numerically. We carry out this simulation work for average feed Reynolds numbers in the laminar flow regime and a permeate flow condition at the membrane surface.

## 2. Materials and Methods

**Figure 2:**
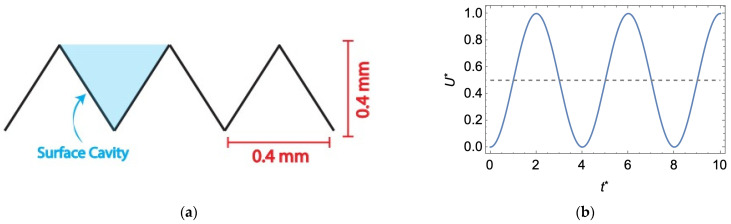
(**a**) Triangular membrane surface pattern studied in this work; (**b**) the sinusoidal waveform used to modulate the average inlet flow velocity, U, where * denotes a dimensionless variable—see Equation (7).

In this study, we replicate the simulation parameters utilized by Won et al. [32], namely, a two-dimensional isosceles triangle prism pattern with a height and width of 400 μm (pictured below in Figure 2 and labeled pattern B in their work), a crossflow feed velocity with a Reynolds number of 600 and 1600, and a constant permeate flow rate (i.e., membrane flux) that is 1/2000 of the maximum feed flow velocity (as specified in Equation (5)). We modulate the feed flow via a sinusoidal waveform with a dimensionless period of 4 and an amplitude of 0.5; as provided in Figure 2, dimensionless parameters are denoted by an asterisk. While the choice in waveform shape is somewhat arbitrary, we note the ability of the sinusoidal waveform to maintain a higher permeate flux over other waveform shapes, as demonstrated by Li et al. [61] in a study on the fouling of hollow fiber membranes subject to pulsed feed flows.

In order to approximate the trajectories of particles in the feed solution, we integrate the simulated velocity field, given an initial starting position for each particle. This method does not account for particle mass or physical/chemical characteristics, interactions with other particles in the feed solution, or with the membrane surface.

### 2.1. Numerical Solver

The numerical solver used in this work is the same as the solver described by Young and Kabala [53,62], which we provide a brief overview of below. First, we use Mathematica’s numerical differential equation solver, *NDSolve*, to solve the incompressible form of the continuity (mass conservation) and Navier–Stokes (momentum evolution) equations in two dimensions (Equations (1) and (2), respectively):

(1) ∇⋅u=0

(2) $$\rho \left(\frac{\partial u}{\partial t}+u\left(\nabla \cdot u\right)\right)=-\nabla P+\mu \nabla^{2}u$$

Slight modifications to the solver allow for solution in the (dimensionless) time domain (i.e., retention of the local acceleration term in the Navier–Stokes equations). The use of these simplified forms requires that we also greatly simplify the chemistry and complexity of real feed streams; in this work, we assume the properties of water at standard conditions.

At the domain boundaries, we apply a set of Dirichlet boundary conditions to specify a fully developed laminar flow profile at the channel inlet, no slip and no penetration along the top channel boundary, a constant permeation rate along the membrane surface, and outflow via a constant pressure condition (see Equations (3)–(6), respectively). For simplicity, we enforce the permeate flux condition purely in the vertical direction. We specify the inlet velocity profile using the Hagen-Poiseuille model for fully developed channel flow.

(3) $$u\left(t,0,y\right)=-\frac{h^{2}}{2\mu }\frac{dP}{dx}\left[\frac{y}{h}\left(1-\frac{y}{h}\right)\right],v\left(t,0,y\right)=0$$

(4) $$u\left(t,x,1\right)=0,\;v\left(t,\;x,\;1\right)=0$$

(5) $$u\left(t,x,y\leq 0\right)=0,\;v\left(t,\;x,y\leq 0\right)=-\frac{1}{2000}u\left(t,0,\;0.5\right)$$

(6) $$P\left(t,\;x=channel\;length,y\right)=0$$

As provided in Equation (7) below, we normalize the system by the cavity depth, *d*, and the average inlet flow velocity, *U*, which we restrict to the laminar flow regime (i.e., Re < 2400). We choose the scaling on the pressure term to be $\rho U^{2}$ given that we anticipate inertial effects to dominate the bulk flow.

(7) $$x^{*}=\frac{x}{d},\;\;y^{*}=\frac{y}{d},\;\;u^{*}=\frac{u}{U},\;\;v^{*}=\frac{v}{U},\;\;t^{*}=\frac{tU}{d},\;\;P^{*}=\frac{P}{{\rho U}^{2}}$$

Finally, because we assume the membrane bore and surface to be fully saturated, we assign flow to the entire domain.

### 2.2. Solver Domain

**Figure 3:**
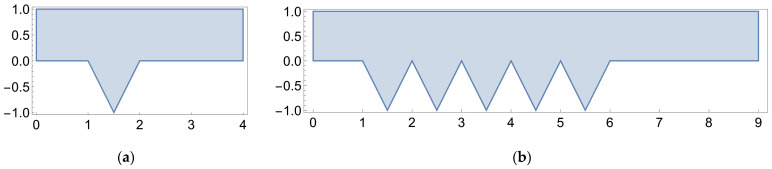
(**a**) Flow geometry for a single cavity geometry; (**b**) sequential cavity geometry.

To idealize the membrane surface, we utilize the single or sequential triangular cavity geometries pictured in Figure 3 (the former to minimize simulation time). In either case, the geometry through-channel extends past the cavity by at least the cavity depth on each side to eliminate end effects from the channel inlet and outlet. Discretization of the flow geometry is accomplished through the use of the *ToElementMesh* function. By default, this function generates a second-order, triangular element mesh. We refine the interior and boundary mesh elements via the *MaxCellMeasure* and *MaxBoundaryCellMeasure* commands. For stream plot generation, we set the *MeshQualityGoal* to 1/1 to capture the details of the cavity vortices. For the geometry pictured in Figure 3a, these parameters yield a mesh with 9.5 × 10^5^ elements with an average quality of 0.91/1. Young and Kabala [53] provide a brief mesh convergence analysis, which we adhere to, in their Supplemental Material.

Here, we call attention to the channel height of the flow geometry, which we set equal to the cavity depth. In reality, we know the former to be much larger than the latter. Even still, the flow geometry pictured above has been successfully used to approximate mass transport across surfaces with grooves and cavities (e.g., Shen and Floryan [54] and Fang et al. [55]). In the study of steady flows over patterned membrane surfaces, other researchers have used truncated domains wherein the channel height is multiple times larger than the cavity depth, and a velocity boundary condition is assigned to the upper channel boundary (e.g., Jung and Ahn [47]). Unfortunately, this boundary condition would be inappropriate to assign to a pulsed flow simulation due to the presence of mixing induced in the bulk flow. Thus, we move forward with the pictured geometry, noting that adjustments should be made to the channel height (relative to the cavity depth) should the aim of the simulation be to replicate experimental results.

## 3. Results

### 3.1. Flow Visualization

#### 3.1.1. Steady Flow

**Figure 4:**
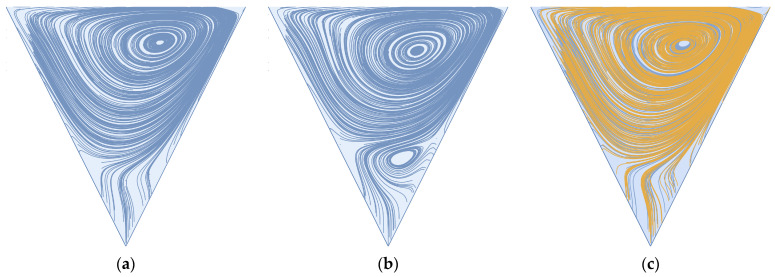
(**a**) Vortex formation in a single cavity exposed to steady flow for Re = 600; (**b**) Re = 1600; (**c**) comparison of stream plot lines in the first (blue) and last (yellow) cavities of sequential cavity geometry (see Figure 3).

We start by replicating the work of Won et al. [32]. In Figure 4, we present cavity stream plots at Reynolds numbers 600 and 1600 and illustrate the relative volumes of the mobile and immobile zones of the membrane surface cavity. As defined by Young and Kabala [53], the mobile zone refers to the volume of cavity space conducive to through-flow, and the immobile zone corresponds to the recirculatory volume in the cavity space. Here, through-flow refers to the portion of feed flow that becomes permeate flow. As demonstrated by Won et al. [32], an increase in Reynolds number results in an increase in the size of the immobile zone. These results are also in agreement with our previous studies on impermeable cavities; see Young and Kabala [53,62]. We note that an increase in Reynolds number results in the generation of a secondary, counter-rotating vortex large enough to be observed without zooming in on a subsection of the cavity space. As previously described by Moffatt [63], we should expect to see an increase in the number of sequential (Moffatt) vortices as we decrease the strength of the permeate flow condition. It is reasonable to postulate that, under steady flow conditions, particulate captured in this secondary vortex will remain in perpetuity due to flow separation between it and the primary cavity vortex caused by the permeate stream (see Figure 4b). For the reader’s edification, we replicate this simulation at a Reynolds number of 600 for a flow domain containing five sequential cavities and compare the stream plots of the first and last cavities (see Figure 4c). We see no significant difference in the stream plots, thus supporting the use of a single idealized cavity space for flow-field imaging and particle tracking.

We note that the steady-state, two-dimensional flow in triangular cavities has already been addressed analytically [63] and numerically [64,65,66]. However, these studies dealt with the driven triangular cavity (i.e., the base of the inverted triangle moves at a specified velocity). In this paper, we deal with flow in open triangular cavities, which allows for interaction between the mobile and immobile zones (as we demonstrate below for pulsed flows).

#### 3.1.2. Pulsed Flow

**Figure 5:**
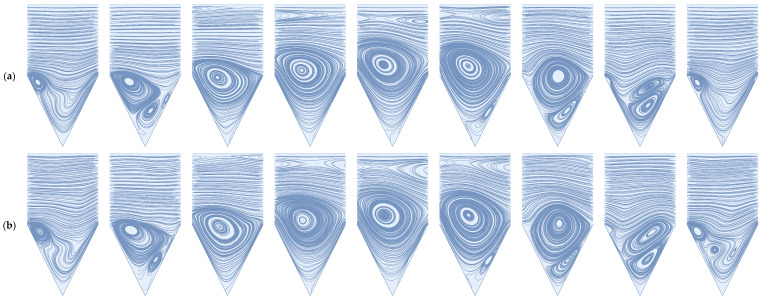
The deep sweep followed by a vortex ejection for a rapidly pulsed feed flow and a permeate flux condition of 1/2000 the maximum inlet flow velocity: (**a**) average flow Re = 600; (**b**) average flow Re = 1600.

**Figure 6:**
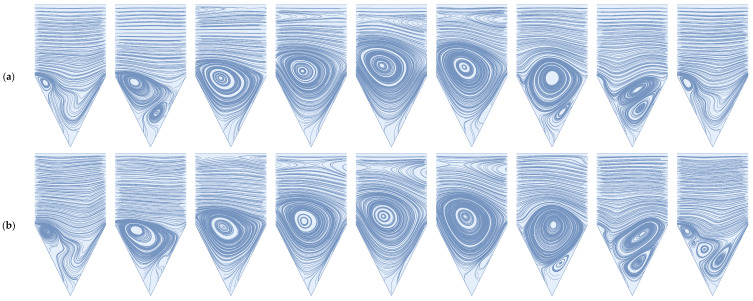
The deep sweep followed by a vortex ejection for a rapidly pulsed feed flow and an increased permeate flux condition of 1/200 the maximum inlet flow velocity; (**a**) average flow Re = 600; (**b**) average flow Re = 1600.

In Figure 5 and Figure 6 below, we illustrate the deep sweep and vortex ejection mechanisms in the cavity space that result from a sinusoidal feed flow with an average Reynolds number of 600 and 1600. The deep sweep and vortex ejection manifest themselves somewhat differently over the patterned membrane surface than they do in the cavity spaces of impermeable media. The high Reynolds numbers of crossflows over membrane surfaces induce additional mixing in the cavity space and, therefore, the generation of secondary vortices comparable in size to the primary cavity vortex. As the through-channel flow volume abruptly increases and decreases, these vortices interact, clouding our ability to easily recognize the deep sweep and the vortex ejection as they occur at lower Reynolds numbers. Despite the presence of these additional vortices, the deep sweep and vortex ejection are still present in the high Reynolds number flows we study in this work, as evidenced by Figure 5 and Figure 6. Readers are again directed to the Supplemental Material to visualize these mechanisms in an impermeable square cavity space at an average Reynolds number of 10. This sequence clearly demonstrates the deep sweep and vortex ejection streamline patterns.

Similar to steady flow conditions, we observe that an increase in Reynolds number results in an increase in immobile zone volume; this is true over the entire period of the inlet waveform. Notably, during the vortex ejection mechanism, we observe the formation of a large counter-rotating vortex along the upper channel wall. In the Supplemental Material, we verify that the existence of this vortex is not strictly an artifact of the selected channel height. For a channel height 10 times that of the pattern depth, the presence of this vortex persists. Therefore, we conclude that the vortex ejection mechanism is not only capable of flushing contaminants from the cavity space but also of inducing large-scale mixing and shear reversals along boundaries within the immediate vicinity of the cavity geometry.

Finally, we confirm the presence of the deep sweep and vortex ejection mechanisms when the magnitude of the permeate flux is increased by a factor of 10. Because these mechanisms have been previously shown to exist in impermeable cavity spaces (e.g., Kahler and Kabala [56] and Young and Kabala [53,62]), we do not decrease the magnitude of the permeate flux condition. A comparison of the stream plot sequences that result from the two flux conditions reveals a decrease in immobile zone volume and an increase in permeate flow volume. It is likely that this decrease in immobile zone volume will result in more particle accumulation at the membrane surface, even in the presence of the deep sweep and vortex ejection mechanisms. Steady-flow simulations provide support for this conclusion. Won et al. [32] found that an increase in the permeation stream area relative to the vortex stream area resulted in a higher degree of surface fouling. Jung and Ahn [47] confirm that an increase in the volume of the “inaccessible” zone results in a decrease in the probability that particles will access the membrane surface. After a review of the past two decades of experimental and numerical data on patterned membrane flows, Wang et al. [51] add further support for this conclusion. 

### 3.2. Particle Tracking

In order to confirm the ability of the deep sweep and vortex ejection mechanisms to remove particles from the cavity space, we visualize the trajectory of massless particles via numerical integration of the velocity field over time.

#### 3.2.1. Steady Flow

**Figure 7:**
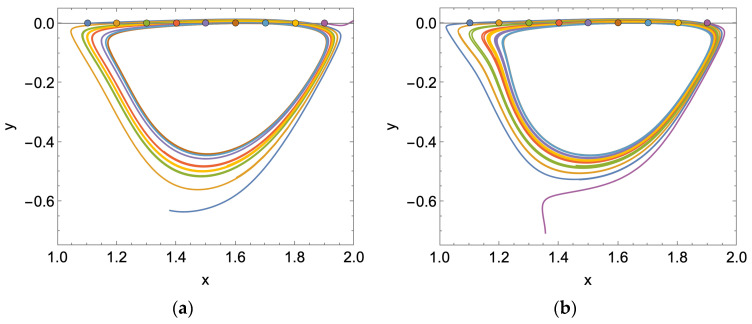
Trajectories of particles uniformly spaced along the boundary of the membrane surface cavity and feed channel, subject to a steady flow: (**a**) Re = 600; (**b**) Re = 1600.

To start, we demonstrate the fate of particles seeded at the geometric boundary between the feed channel and cavity space under steady flow conditions. The results presented in Figure 7 are for a dimensionless simulation time of 100.

**Figure 8:**
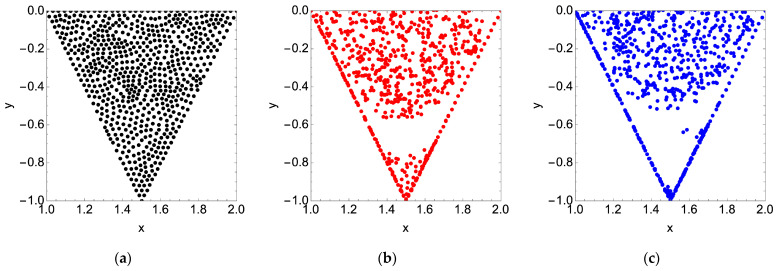
(**a**) Initial arbitrary cavity particle positions; (**b**) final cavity particle positions for a steady feed flow condition and dimensionless simulation time of 200 with Re = 600 (red); (**c**) Re = 1600 (blue).

For a Reynolds number of 600, we observe that all but two of the nine particles end up in the recirculation zone; of the remainder, one particle rejoins the bulk flow, and the other settles at the membrane surface. For a Reynolds number of 1600, the only particle that escapes recirculation also settles at the membrane surface. As expected, if we populate the cavity space with a grid of particles, we observe that the particles remain almost entirely in the cavity space. For a Reynolds number of 600 and 1600, roughly 2% of the particles leave the cavity space and enter the bulk flow. In general, the final location of the particles is the same for each of the tested Reynolds numbers. The results presented in Figure 8 are for a dimensionless simulation time of 200.

**Figure 9:**
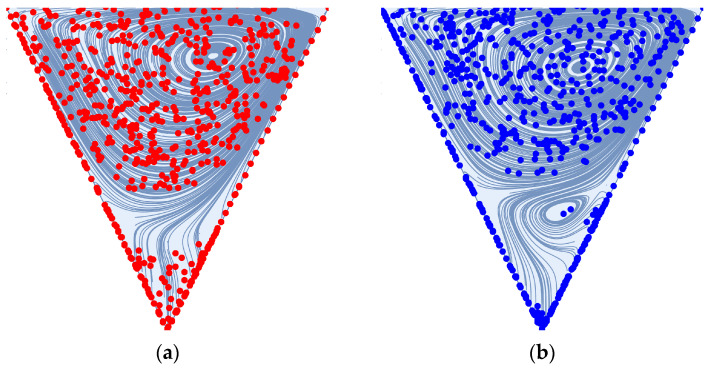
Final cavity particle positions for a steady feed flow condition (see Figure 8) are driven by the locations of the recirculation zones in the membrane surface pattern: (**a**) Re = 600; (**b**) Re = 1600.

To explain the void without particles in the lower third of the cavity space, we refer to the stream plots presented in Figure 4. Overlaying the two plots in Figure 9, we see that the permeation volume remains clear of particles. We also observe a secondary immobile zone on the downstream cavity wall responsible for a small grouping of particles for Re = 1600.

#### 3.2.2. Pulsed Flow

**Figure 10:**
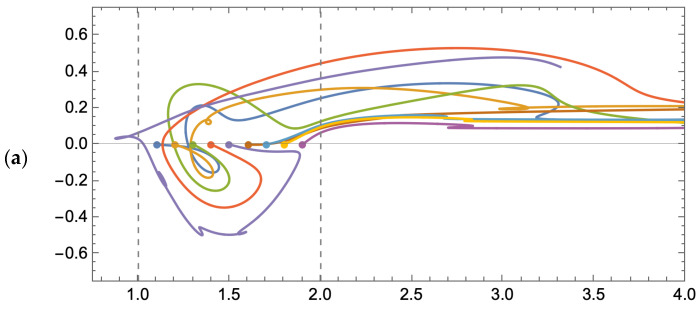

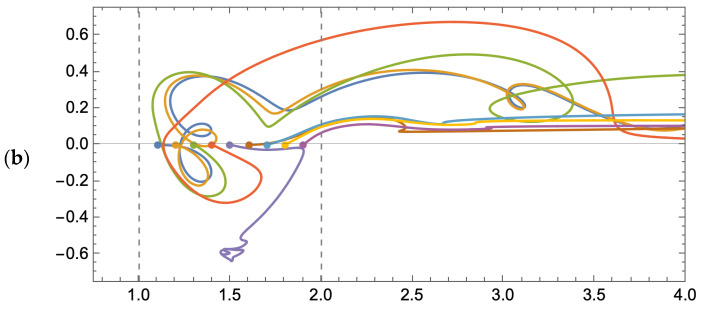
Trajectories of particles uniformly spaced along the boundary of the membrane surface cavity and feed channel, subject to a rapidly pulsed feed flow: (**a**) average Re = 600; (**b**) average Re = 1600.

For a rapidly pulsed inlet flow, we observe that for the boundary particles, all but one particle is swept away into the bulk flow; see Figure 10 below. Although the particles may enter the cavity space, they are not subject to remaining there indefinitely, as is the case for steady flow (refer to Figure 7 for comparison). In order to reduce computational demand, we restrict the dimensionless simulation time to 20.

**Figure 11:**
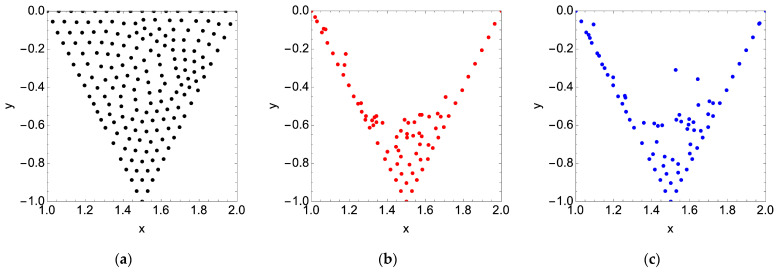
(**a**) Initial arbitrary cavity particle positions; (**b**) final cavity particle positions after 4 pulses of the feed flow with an average Re = 600 (red); (**c**) Re = 1600 (blue).

If we then seed the cavity space with particles, albeit over a coarser grid to minimize computational demand (180 particles versus the 668 used for steady flow simulation), we observe that over half of the particles move into the bulk flow: 57% for an average Reynolds number of 600 and 59% for 1600. The final particle locations in the cavity space are provided in Figure 11 below.

## 4. Discussion

The plots in Figure 11 clearly demonstrate the ability of a rapidly pulsed flow to remove particles from the cavity space via vortex ejection and the deep sweep mechanisms. However, to maximize removal, it remains necessary to optimize the shape of the waveform relative to the pattern morphology, average flow Reynolds number, average particle size, and resonant frequency of the system. This exercise will likely yield situationally specific results. For example, while Nishimura et al. [67] found a pronounced dependence on waveform amplitude or oscillatory fraction at low Reynolds numbers, Greiner [68] found a significant dependence on frequency at moderate Reynolds numbers. Future analysis should be based on the work of Nishimura et al. [67,69] and Greiner [68], García-Picazo et al. [28], and Kahler and Kabala [56], who optimized waveform parameters in periodically grooved channels, flat sheet membranes with spacers, and granular media, respectively. Reference should also be given to Li et al. [61], who determine the effect of waveform shape on surface fouling. Further, processing conditions such as temperature and transmembrane pressure must also be considered, as recently demonstrated for non-patterned membranes by Aloulou et al. [70]. Despite the need for optimization, the arbitrary waveform selected for this study still removes approximately 30 times more particles from the cavity space in roughly 1/10th of the time, supporting our earlier statement that this type of inlet flow should reduce the need for and frequency of traditional cleaning methods.

Finally, it remains prudent to discuss the accuracy of the presented results. Aside from the numerical error associated with the simulation parameters (e.g., mesh size, precision and accuracy goals, time-step, solver method, etc.), the accuracy of our results is limited by a few simplifying assumptions. Namely, we do not account for the surface roughness of the membrane, which would induce local turbulence. We also enforce a uniform permeate flux purely in the vertical direction, violating the no-slip condition. Despite the fact that the no-slip condition generally does not apply at the boundary between a porous material and a free-flowing fluid [71,72], as highlighted by Belfort and Nagata [73], it is commonly applied to such scenarios (e.g., the simulation off of which we build our work: Won et al. [32]). Further, as the membrane fouls, the flux condition would be non-uniform along its length due to various factors, such as an increase in boundary layer thickness and a reduction in transmembrane pressure. Finally, and most significantly, we simulate only the flow of massless particles, meaning that we neglect interactions between the particles and the physical and chemical properties of the membrane itself. Nonetheless, the results provided in this study reveal an accurate first-order representation of the flow field induced over a patterned membrane surface subject to a rapidly pulsed flow.

## 5. Conclusions

In this work, we demonstrate the ability of a rapidly pulsed feed flow to remove particles trapped in the cavity-like spaces of patterned membrane surfaces (i.e., valleys and channels). When subject to a steady feed flow, particles become trapped in recirculation zones of cavity-like spaces, where they aggregate and aggravate surface fouling. A rapidly pulsed flow induces the deep sweep and vortex ejection mechanisms and flushes recirculation zones with a relatively clean flow volume. This work is well-suited for further expansion, especially considering its application to fouling prevention. It is necessary to identify how the pulse frequency and amplitude affect the ability of these mechanisms to move particles away from the membrane surface. The results of this work suggest that when combined with a rapidly pulsed inlet flow, patterned membrane surfaces can not only alleviate concentration polarization and the surface fouling that follows but also reduce the need for traditional cleaning methods that require operational downtime and often involve the use of abrasive chemical agents.

## Data Availability

The data presented in this study are openly available at: https://osf.io/2hry8/?view_only=568d53149699485c834d64201810ad25.

## Supplementary Materials

The following supporting information can be downloaded at: https://www.mdpi.com/article/10.3390/membranes14010021/s1, The video file, entitled “DEP_CavityAnimation.mp4” demonstrates the deep sweep and vortex ejection mechanisms in sequence in an impermeable cavity.
